# Efficacy Evaluation of a Combined Hemorrhagic Septicemia–Mastitis Vaccine in Dairy Cows and Buffaloes

**DOI:** 10.3390/ani12060706

**Published:** 2022-03-11

**Authors:** Qudratullah, Ghulam Muhammad, Tariq Jamil, Imaad Rashid, Qudrat Ullah, Muhammad Saqib

**Affiliations:** 1 Department of Surgery, Cholistan University of Veterinary & Animal Sciences, Bahawalpur 63100, Pakistan; qudratullah@cuvas.edu.pk; 2 Department of Clinical Medicine and Surgery, Faculty of Veterinary Science, University of Agriculture, Faisalabad 38000, Pakistan; profdrgm54@gmail.com (G.M.); imaad.rasheed@uaf.edu.pk (I.R.); 3 Institute of Bacterial Infections and Zoonoses, Friedrich-Loeffler-Institut, 07743 Jena, Germany; 4 Faculty of Veterinary and Animal Sciences, The University of Agriculture, Dera Ismail Khan 29111, Pakistan; qudratmahsud@gmail.com

**Keywords:** HS, mastitis, immunogenicity, Montanide^®^, ELISA, somatic cell count

## Abstract

**Simple Summary:**

Hemorrhagic septicemia (HS) and mastitis are important diseases of South Asian dairy animals. Seventy *S. aureus*/*Str. agalactiae*-free lactating buffaloes (n = 45) and cows (n = 25) and fifty *S. aureus*/*Str. agalactiae*-positive lactating (early stage of lactation) buffaloes (n = 25) and cows (n = 25) were subjected to two doses of Montanide^®^ adjuvant combined HS–mastitis vaccine with 21 days of interval. Vaccinated groups showed mean somatic cell counts and mastitis severity scores that were significantly lower (*p* < 0.05), whereas the milk yield was significantly higher (*p* < 0.05). In conclusion, this vaccine can be used as a potential preventive measure against HS and mastitis in dairy animals.

**Abstract:**

Hemorrhagic septicemia (HS) and mastitis caused by *Pasteurella (P.) multocida*, *Staphylococcus (S.) aureus* and *Streptococcus (Str.) agalactiae* are important ailments of the dairy industry especially in South Asia. The present study evaluates the efficacy of a locally prepared hemorrhagic septicemia (HS) and mastitis combined vaccine. To this end, a total of 70 HS, *S. aureus* and *Str. agalactiae*-free lactating (early stage of lactation) buffaloes (n = 45) and cows (n = 25), and 50 lactating (early stage of lactation) cows (n = 25) and buffaloes (n = 25) positive for *S. aureus*/*Str. agalactiae* were subjected to two doses of HS–mastitis combined vaccine with an interval of 21 days. Antibody response was monitored by ELISA up to six months (180 days). Antibody titers against HS and mastitis were significantly (*p* ˂ 0.05) higher in vaccinated groups as compared to the non-vaccinated groups. Cumulative mean somatic cell counts and mastitis severity scores in vaccinated groups were significantly lower (*p* < 0.05), and milk yield was significantly higher (*p* < 0.05) than those in the respective non-vaccinated controls. In conclusion, Montanide^®^-adjuvanted HS–mastitis combined vaccine showed significant immunogenic effects in dairy cows and buffaloes. However, challenge studies remain overdue.

## 1. Introduction

Hemorrhagic septicemia (HS) and bacterial mastitis are important ailments in South Asian dairy animals [1,2,3,4]. Both diseases lead to serious economic losses in terms of mortality, production loss and treatment costs directly on farmers, whereas surveillance programs cost additional burden on the national economy [1]. Hemorrhagic septicemia is an infectious, fatal bacterial disease caused by *P. multocida* type B:2 [5] which accounts for annual losses of USD 12.4 million (as of 2022) in the province of Punjab only. The HS has been ranked first among the economically important diseases of livestock in Pakistan and approximately 50% reduction in the incidence of this disease has been estimated viable to bridge the gap between milk demand and supply [6]. Mastitis (inflammation or swelling of milk-producing organ) is another common dairy animal disease which, although not fatal, causes colossal economic losses to our resource-poor dairy farmers and milk-processing industry [7]. Mastitis-affected populations of buffaloes and cattle not only sustain ~25% reduction in their milk yield but also render the milk unwholesome for human consumption as it may contain pathogenic bacteria, toxins and other harmful substances which may not be neutralized by ultra-high temperature (UHT) treatment [7].

Mastitis preventive measures are not strictly followed and contagious mastitis remains quite rife in dairy animals of Pakistan [8]. Of the contagious mastitogens, *Staphylococcus (S.) aureus* and *Streptococcus (Str.) agalactiae* are the major pathogens in Pakistani dairy cows and buffaloes [8,9,10,11]. Control of such diseases which are of the mammary gland and cause systemic health concerns is therefore imperative for economically viable dairy farming as well as to produce quality milk. The aim of this study was to evaluate the field effectiveness of a combined HS–mastitis vaccine in dairy cows and buffaloes.

## 2. Materials and Methods

### 2.1. Origin and Selection of the Vaccinal Isolates

Peripheral blood and bone marrow samples from clinical cases of HS were cultured on blood, casein-sucrose-yeast (CSY) and MacConkey’s agar for isolation of *P. multocida*; quarter foremilk samples from clinically affected cows and buffaloes were used for the isolation of *S. aureus* and *Str. agalactiae* as per the National Mastitis Council (NMC), New Prague, Minnesota, USA guidelines [12]. Presumptive isolates were biochemically typed and confirmed by polymerase chain reaction (PCR) followed by in vivo virulence and immunogenicity testing before deciding to select the candidate isolates [13,14].

### 2.2. Preparation of HS–Mastitis Combined Vaccine

The selected vaccine isolates were grown independently in nutrient broth supplemented with 10% sterile bubaline whey at 37 °C for 48 h. It was inactivated by adding (0.4% v/v) 37% formaldehyde and incubating at 4 °C for 1 h. Finally, Montanide^®^ ISA 201 VG (Seppic, Courbevoie, France), sodium azide and thimerosal were added to make a volume of 5 mL/dose of vaccine containing 5 × 10^9^ cells of each *P. multocida*, *S. aureus* and *Str. agalactiae* as described previously in detail [15].

### 2.3. Preliminary Control-Testing of the Combined Vaccine

Sterility, safety and preliminary efficacy-control testing was performed by culturing 20 μL of the final combined vaccine volume on blood and MacConkey agar plates (incubated at 37 °C for 48 h), intramuscular administration of 10 mL (double of the recommended dose) vaccine into four bovine/bubaline and 0.2 mL into five murine models and observed for fourteen days for any adverse reaction and 0.1 mL intraperitoneal administration of the final combined vaccine at fifteen days interval followed by an intraperitoneal challenge of the vaccinal isolates into murine models [14]. Finally a stability testing of the combined vaccine was performed by incubating the vaccine vials and recording the stability parameters (e.g., pH, sedimentation, color and texture of the vaccine) at 4 °C for six months [14].

### 2.4. Experimental Design

A total of 70 lactating (early stage of lactation) non-infected (including 45 buffalo and 25 cows) and 50 infected lactating (early stage of lactation) animals, including 25 buffaloes and 25 cows were used for evaluation of the HS–mastitis combined vaccine. The animals selected were raised at the Livestock Experimental Station (LES), University of Agriculture, Faisalabad, Pakistan, Ayub Agriculture Research Institute (AARI), Faisalabad, Pakistan and two private dairy farms at Faisalabad, Pakistan. There was no history of vaccination against HS for the last six months on these animals and this was further confirmed by negative ELISA results as mentioned under subheading 2.5. All animals were of local breeds and were raised semi-intensively in covered-shed and an open area. They were mostly fed with seasonal fodders depending on calculated intake of dry matter contents and concentrate supplements. Fresh water was fed *ad libitum*.

These animals were divided into four groups, i.e., in Group A, B, C and D. In Group A, non-infected (non-mastitic) animals were treated with HS–mastitis combined vaccine, Group B animals served as normal control, Group C animals included mastitic animals treated with HS–mastitis combined vaccine against *P. multocida*, *S. aureus* and *Str. agalactiae* and Group D contained mastitic animals serving as infected control as shown in Table 1. All animals were evaluated for six months by ELISA against anti- *P. multocida*, *S. aureus* and *Str. agalactiae* antibodies, somatic cell count, severity of mastitis and milk yield, as described under 2.5.

### 2.5. Evaluation Parameters

An in-house indirect ELISA was performed to determine antibody titers against *P. multocida*, *S. aureus* and *Str. agalactiae* from 20% of randomly selected vaccinated and control animals at day 60, 120 and 180 post vaccination [15,17,18]. Briefly, ELISA plates were coated with bicarbonate buffer (Sigma, Burlington, YT, USA) containing 15 µg antigen/mL. The plates were blocked with bovine serum albumin (0.1%) and antibodies detected by protein-G HRP conjugate (LSI Vet, Lissieu, France). Finally, TMB (3, 3″, 5, 5″-Tetramethylbenzidine; Abcam, Cambridge, UK) was used as substrate and 1M sulfuric acid as stop solution. Optical density was measured at 450 nm [14].

Somatic cell count was monitored in vaccinated and control groups at day 60, 120 and 180 post vaccination. Briefly, 10 μL of milk sample was spread and dried on a glass slide at 30–40 °C. Following defatting (submerging the slides into xylene for 1–2 min) and drying, the slides were stained with Newman–Lampert stain for 15 min (supplemented by blue aliquot of Diff Quick Stain (Difco Labs., Detroit, MI, USA) for 10–15 s), rinsed and dried. Cells were counted at 1000× in 50 fields which were multiplied by microscopic factor (MF) to obtain the number of cells per ml of milk. The number was then multiplied by 1000 to calculate the number of somatic cells/mL of milk [19,20].

The severity of clinical cases of mastitis in vaccinated, non-vaccinated and control groups was determined as per [21]. Briefly, visible changes in milk (clots, neutrophils, etc.) without clinical illness of udder and cow were classified as Grade 1, whereas visible milk changes accompanied with visible udder illness without clinical illness of cow were classified as Grade 2 A (acute). Grade 2 B, if the quarter was hard and lumpy but not painful (may be charged or contracted = ‘high’), the mastitis is chronic. Grade 3 = a quarter with a 2 A grade and the cow was ill.

Mean milk yield was determined at day 60, 120 and 180 post vaccinations in liters per 24 h.

### 2.6. Statistical Analysis

The data collected were subjected to statistical analysis using ANOVA and other appropriate design software packages (SAS Version 9.1, SAS Institute Inc., Cary, NC, USA). Mammary quarters were considered as unit of concern for prevalence, incidence and somatic cell count. *p* ≤ 0.05 was considered as level of significance.

## 3. Results

### 3.1. Serum Antibody Titers against Selected Pathogens

The highest anti-*P. multocida* IgG values (OD 2.13), anti-staphylococcal IgG values (OD 2.56) and anti-*Str. agalactiae* IgG values (OD 1.88) were recorded in Group A at day 60, and they remained significantly higher throughout the observation period (180 days) than pre-vaccination titer (day zero), as presented in Table 2.

The result of present study revealed that the administration of Montanide^®^-adjuvanted HS–mastitis combined vaccine produced higher antibody titers against *P. multocida, S. aureus* and *Str. agalactiae* in the vaccinated group (A and C) compared to the non-vaccinated groups (B and D). The antibody titers were higher in non-diseased vaccinated (Group A) animals compared to diseased vaccinated animals (Group C) at 0, 60, 120 and 180 days post vaccination, as is evident in Table 2. It was observed that the antibody titers against *P. multocida, S. aureus* and *Str. agalactiae* augmented at 60 and 120 days of vaccine administration in healthy vaccinated animals and decreased at 180 days post vaccination (Table 2). All the vaccinated groups showed significant effect (*p* < 0.05) on antibody titers when compared with non-vaccinated groups.

### 3.2. Milk Somatic Cell Count

**Figure 1:**
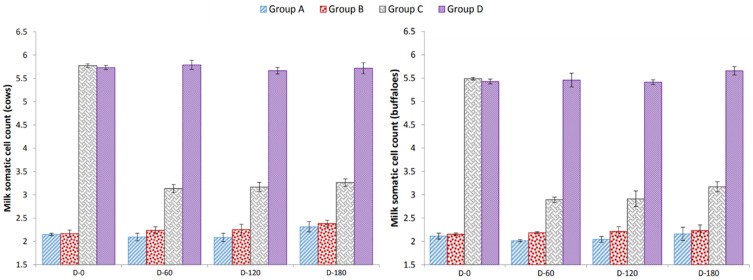
Effect of HS–mastitis combined vaccine on somatic cell count (×10^5^ mL of milk); Group A = vaccinated cows (Milk culture − ve for *S. aureus* and *Str. agalactiae*, SFMT − ve); Group B = unvaccinated cows (Milk culture − ve for *S. aureus* and *Str. agalactiae*, SFMT − ve); Group C = vaccinated cows (Milk culture + ve for *S. aureus* and *Str. agalactiae*, SFMT + ve); Group D = unvaccinated cows (Milk culture + ve for *S. aureus* and *Str. agalactiae*, SFMT + ve).

A non-significant decrease in somatic cell count in milk during experimental days was observed in Groups A, B and D. However, a significant decrease in somatic cell count was observed in Group C after 60 days of vaccination (Figure 1).

### 3.3. Severity of Mastitis in Vaccinated and Unvaccinated Animals

Table 3 shows that the severity scores of Group A were lower than those of Group B. Similarly, mean of the severity scores of Group C were lower than those of Group D.

### 3.4. Effect of Vaccination on Milk Yield

Statistical analysis (ANOVA) did not show overall any significant difference (*p* > 0.05) in milk yield of all groups. During comparison of vaccinated groups with non-vaccinated groups by Tukey’s test (ANOVA), it was observed that at day 60, milk yield was increased significantly (*p* < 0.01) in vaccinated animals and decreased in non-vaccinated animals except for the buffaloes in Group B. There was a slight increase, but not at a significant level (*p* > 0.05), in these buffaloes. At day 120 post vaccination, all groups showed a decrease in milk when compared with milk yield at day 60, which was further decreased significantly (*p* < 0.001) at day 180 in all groups (Table 4).

## 4. Discussion

A high degree of immunogenicity is one of the cardinal considerations in selecting an isolate for vaccine production. There is always room for a safe, effective and polyvalent vaccine [5,22]. Previously, high-antibody titers were observed in animals of all age groups vaccinated with the oil-adjuvanted vaccine as compared to the animals vaccinated with alum precipitated vaccine, i.e., the titers declined after three months and reached minimal levels at the 180th day post vaccination [23]. Hence there was a need to look for a replacement of the adjuvant that could provide protection for a longer period of time. Therefore, Montanide^®^ ISA 201 VG was tested as an adjuvant in this study.

The preventive and curative attributes of this vaccine found in our study, i.e., serum antibody titers, somatic cell count, severity of mastitis and milk yield agreed with previous studies [24,25]. Antibody titers against *P. multocida*, *S. aureus* and *Str. agalactiae* augmented at 60 and 120 days of vaccine administration in non-diseased vaccinated animals and decreased at 180 days post vaccination; this result was congruent with the findings from previous studies [24]. A non-significant decrease in somatic cell count was noted at day 60, and day 120 in vaccinated non-diseased cows and buffaloes. At day 180 post vaccination, somatic cell count in this group was non-significantly higher than at days 0, 60 and 120. In non-vaccinated non-diseased cows and buffaloes, a non-significant increase in somatic cell count was recorded at days 60, 120 and 180. Vaccination of mastitic cows and buffaloes caused a significant decrease in somatic cell count at all post-vaccination observation time points (days 60, 120 and 180), albeit at day 180, a slight increase in somatic cell count was noticed. Somatic cell counts in non-vaccinated diseased cows and buffaloes differed non-significantly at days 0, 60, 120 and 180. These somatic cell counts at the 28th day post treatment are consistent with Owens et al. [26]. In addition, mean ± SEM of the group quarter mastitis severity scores were lower in vaccinated groups (Group A cows = 0.125 ± 0.0639, Group A buffaloes = 0.025 ± 0.0186; Group C cows = 0.05 ± 0.0402, Group C buffaloes = 0.033 ± 0.0234) than in unvaccinated groups (Group B cows = 0.20 ± 0.0186, Group B buffaloes 0.12 ± 0.0481; Group D cows = 0.20 ± 0.0859, Group D buffaloes = 0.15 ± 0.0706). In this respect, the results of the present study are in conformity with previous results [10,17,24]. Contrarily, our results conflict with those of earlier findings [27]. These investigators determined the effect of an aluminum hydroxide adjuvant autogenous *S. aureus* bacterin on the prevalence of *S. aureus*, somatic cell count and clinical mastitis in a dairy herd beset with infections with this organism. Animal and quarter-based prevalence of *S. aureus* did not differ between cows treated with autogenous vaccine or those treated with a placebo. Although the HS–mastitis combined vaccine tested in the present study displayed both preventive and curative effect on mastitis, its use should be combined with standard mastitis control measures such as antiseptic teat dipping, dry cow therapy and culling of chronically infected animals. The increase in milk yield of animals treated with HS–mastitis combined vaccine in the present study is consistent with the findings of [24,28]. Nevertheless, in view of the limited number of tested animals, a large-scale trial of the vaccine is necessary before utilization at a mass scale. Furthermore, the trails must include exotic/high-yielding animals for a broader idea of the efficacy results of the vaccine.

Montanide^®^ are ready-to-use mineral oil adjuvants and are cheaper in use. Montanide^®^-based mastitis vaccine previously did induce higher immune responses against α and β toxins of *S. aureus* in sheep and cattle [18,29]. Montanide^®^ adjuvants are less viscous and more tolerable to animals than traditional Freund’s adjuvant [30]. Montanide^®^ ISA 201 VG adjuvant quadrivalent foot-and-mouth vaccine has been reported to elicit a rapid and longer immune response compared to that elicited by aluminum hydroxide gel [31]. Owing to these desirable attributes, the HS–mastitis combined vaccine tested in the present study can be utilized as a preventive measure.

## 5. Conclusions

Montanide^®^-adjuvanted HS–mastitis combined vaccine incorporating *S. aureus* and *Str. agalactiae* (the two most prevalent mastitis pathogens in Pakistan as reviewed by [10]) displayed both preventive and curative qualities. Montanide^®^-adjuvanted HS–mastitis combined vaccine can be used effectively for HS and mastitis prevention caused by *P. multocida*, *S. aureus* and *Str. agalactiae* in dairy cows and buffaloes. Nevertheless, studies including field isolate-challenge, and larger-scale trials remain overdue. HS and mastitis control measures should be adapted additionally.

## Figures and Tables

**Table 1:** Experimental design.

Groups	Experimental Treatment	Dairy Species and Number (n) of Animals	HS–Mastitis Status at the Time of Initiation of Trial
A	Vaccination twice at 21-day interval	Buffalo (n = 30) Cow (n = 10)	Milk culture − ve for *S. aureus* and *Str. agalactiae*, SFMT − ve; no history of vaccination against HS during the last six months, no or low antibody titers against *P. multocida*
B (control)	No vaccination	Buffalo (n = 15) Cow (n = 15)	Milk culture − ve for *S. aureus* and *Str. agalactiae*, SFMT − ve; no history of vaccination against HS during the last six months, no or low antibody titers against *P. multocida*
C (Sub-clinically mastitic buffaloes and cows)	Vaccination twice at 21-day interval	Buffalo (n = 15) Cow (n = 15)	Milk culture + ve for *S. aureus*/*Str. agalactiae*, SFMT + ve in one or more quarter but no clinical signs of mastitis
D (Sub-clinically mastitic buffaloes and cows) Infected control	No vaccination	Buffalo (n = 10) Cow (n = 10)	Milk culture + ve for *S. aureus*/*Str. agalactiae*, SFMT + ve in one or more quarter but no clinical signs of mastitis

SFMT = Surf field mastitis test (as performed according to [16]).

**Table 2:** Serum antibody titers produced by HS–mastitis combined vaccine.

Type of Antibody Determination	Sampling Day	Group A	Group B	Group C	Group D
anti-*P. multocida*	0	0.47 ± 0.006 ^b^	0.35 ± 0.005 ^d^	0.59 ± 0.004 ^a^	0.41 ± 0.007 ^c^
	60	2.55 ± 0.027 ^a^	0.31 ± 0.011 ^d^	1.63 ± 0.006 ^b^	0.36 ± 0.006 ^c^
	120	2.13 ± 0.013 ^a^	0.31 ± 0.015 ^c^	0.74 ± 0.005 ^b^	0.29 ± 0.006 ^d^
	180	1.31 ± 0.009 ^a^	0.20 ± 0.005 ^d^	0.70 ± 0.005 ^b^	0.30 ± 0.004 ^c^
anti-*S. aureus*	0	0.38 ± 0.007 ^b^	0.36 ± 0.005 ^c^	1.42 ± 0.010 ^a^	0.31 ± 0.007 ^d^
	60	2.56 ± 0.015 ^a^	0.41 ± 0.006 ^c^	1.73 ± 0.006 ^b^	0.39 ± 0.007 ^d^
	120	2.14 ± 0.011 ^a^	0.32 ± 0.007 ^c^	0.84 ± 0.007 ^b^	0.21 ± 0.006 ^d^
	180	1.32 ± 0.008 ^a^	0.30 ± 0.007 ^c^	0.80 ± 0.006 ^b^	0.30 ± 0.006 ^c^
anti-*Str. agalactiae*	0	0.37 ± 0.007 ^b^	0.27 ± 0.005 ^c^	0.51 ± 0.004 ^a^	0.21 ± 0.006 ^d^
	60	1.86 ± 0.009 ^a^	0.32 ± 0.006 ^b^	1.87 ± 0.008 ^a^	0.29 ± 0.007 ^c^
	120	1.88 ± 0.005 ^a^	0.49 ± 0.006 ^c^	1.40 ± 0.006 ^b^	0.19 ± 0.005 ^d^
	180	0.56 ± 0.005 ^b^	0.45 ± 0.005 ^c^	0.80 ± 0.005 ^a^	0.31 ± 0.006 ^d^

Means followed by different letters in a row or in a column are statistically significant (*p* ˂ 0.05) with type of antibody titers determinant (a = highest value followed by b, c and d = lowest value); A = vaccinated cows and buffaloes (Milk culture − ve for *S. aureus* and *Str. agalactiae*, SFMT − ve); B = unvaccinated cows and buffaloes (Milk culture − ve for *S. aureus* and *Str. agalactiae*, SFMT − ve); C = vaccinated cows and buffaloes (Milk culture + ve for *S. aureus* and *Str. agalactiae*, SFMT + ve); D = unvaccinated cows and buffaloes (Milk culture + ve for *S. aureus* and *Str. agalactiae*, SFMT + ve).

**Table 3:** Effects of HS–mastitis combined vaccine on the severity of mastitis.

Animal Groups	Number of Animals	Mean ± SEM * of the Group Quarter Mastitis Severity Scores **	Distribution of Quarters as per Their Mastitis Severity Scores
Group A = vaccinated (SFMT and culture − ve)	cows (n = 10)	0.125 ± 0.0639	3 quarters with score = 1 1 quarter with score = 2
	buffaloes (n = 30)	0.025 ± 0.0186	1 quarter with score = 1 1 quarter with score = 2
Group B = control (SFMT and culture − ve)	cows (n = 15)	0.20 ± 0.0744	5 quarters with score = 1 2 quarters with score = 2 1 quarter with score = 1
	buffaloes (n = 15)	0.12 ± 0.0481	5 quarters with score = 1 1 quarter with score = 2
Group C = vaccinated (SFMT and culture + ve)	cows (n = 15)	0.05 ± 0.0402	2 quarters with score = 1 1 quarter with score = 2
	buffaloes (n = 15)	0.033 ± 0.0234	2 quarters with score = 1
Group D = control (SFMT and culture + ve)	cows (n = 10)	0.20 ± 0.0859	6 quarters with score = 1 2 quarters with score = 2
	buffaloes (n = 10)	0.15 ± 0.0706	5 quarters with score = 1 1 quarter with score = 2

* Aggregate of the quarter severity scores divided by the total of quarters in the respective groups; ** As per Roberson (2003). SFMT = Surf field mastitis test; A = vaccinated cows and buffaloes (Milk culture − ve for *Str. agalactiae* and *S. aureus* SFMT − ve); B = unvaccinated cows and buffaloes (Milk culture − ve for *Str. agalactiae* and *S. aureus*, SFMT − ve); C = vaccinated cows and buffaloes (Milk culture + ve for *S. aureus* and *Str. agalactiae,* SFMT + ve); D = unvaccinated, diseased cows and buffaloes (Milk culture + ve for *S. aureus* and *Str. agalactiae*, SFMT + ve).

**Table 4:** Milk yield (Mean ± SE; L/24 h) of vaccinated and control groups at different post-vaccination days.

Animal Groups	Number of Animals	Milk Yield (Mean ± SE; L/24 h)			
		Day 0	Day 60	Day 120	Day 180
A (Healthy vaccinated)	Cows (n = 10)	7.89 ± 0.15	8.17 ± 0.12↑ ***	7.95 ± 0.17↓	5.45 ± 0.16↓ ***
	Buffaloes (n = 30)	9.13 ± 0.05	9.71 ± 0.06↑ **	9.52 ± 0.03↓	6.22 ± 0.10↓ ***
B (Healthy control)	Cows (n = 15)	7.87 ± 0.19	7.72 ± 0.11↓ *	7.67 ± 0.20↓	4.49 ± 0.22↓ ***
	Buffaloes (n = 15)	9.10 ± 0.18	9.13 ± 0.09↑	8.57 ± 0.31↓	5.15 ± 0.36↓ ***
C (Infected vaccinated)	Cows (n = 15)	5.21 ± 0.13	6.13 ± 0.19↑ **	6.01 ± 0.14↓	4.76 ± 0.25↓ *
	Buffaloes (n = 15)	6.11 ± 0.25	7.10 ± 0.18↑ ***	6.43 ± 0.28↓	4.72 ± 0.23↓ ***
D (Infected control)	Cows (n = 10)	5.18 ± 0.12	5.10 ± 0.08↓	4.95 ± 0.17↓	3.32 ± 0.11↓ ***
	Buffaloes (n = 10)	6.17 ± 0.14	5.86 ± 0.13↓ ***	5.27 ± 0.04↓	3.79 ± 0.12↓ ***

Comparison of groups at different time interval (days) by ANOVA: * = *p* < 0.05, ** = *p* < 0.01, *** = *p* < 0.001; ↓= decrease in milk yield, ↑ = increase in milk yield.

## Data Availability

Not applicable.
